# History, Hype, and Responsible Psychedelic Medicine: A Qualitative Study of Psychedelic Researchers

**DOI:** 10.1007/s11673-024-10386-4

**Published:** 2024-10-04

**Authors:** Michaela Barber, John Gardner, Adrian Carter

**Affiliations:** 1 School of Psychological Sciences, Monash University, Clayton, VIC 3800 Australia; 2 Monash Bioethics Centre, Monash University, Clayton, VIC 3800 Australia

**Keywords:** Psychedelics, Responsible research and innovation, Ethics, Qualitative, Mental health

## Abstract

*Background *Psychedelic medicine is a rapidly growing area of research and policy change. Australia recently became the first country to legalize the prescription of psychedelics and serves as a case study of issues that may emerge in other jurisdictions. Despite their influence as a stakeholder group, there has been little empirical exploration of psychedelic researchers’ views on the development of psychedelic research and the ethical concerns. *Methods* We thematically analysed fourteen interviews with Australian psychedelic researchers. *Results* Three themes were constructed from the data: 1) coming out of the shadow of the 1960s, 2) challenges and affordances in engaging stakeholders, and 3) growing pains in innovation and translation. *Conclusion* The results illustrated tensions arising from the rapid growth of psychedelic research from a small group of dedicated individuals with a similar worldview, to a multi-interest, regulated industry. Participants’ experiences and viewpoints were influenced by the history of psychedelic research, and this was met with an overarching concern for protecting the field from premature discontinuation, as well as maximizing potential positive impacts. Targets for stakeholder collaboration and initiatives to support responsible innovation in psychedelics include equitable access, sustainable industry involvement, productive research agendas, responsible reporting of evidence, and risk-taking within the relative safety of clinical trials.

## Introduction

Psychedelic medicine has a history of promise but also of political backlash (e.g., Nixon’s “War on Drugs”), research abuses (Strauss, de la Salle, and Sloshower 2021), and a lack of reproducible findings (Hall and Farrell 2021) that contributed to the cessation of international research programmes. The contemporary re-emergence of psychedelic medicine has also been promising but carries some of the same concerns (McNamee, Devenot, and Buisson 2023; Aday et al. 2022), and as such there is an urgent need to understand what makes for sustainable, responsible application of psychedelics in medicine. In 1943, the discovery of the psychoactive properties of lysergic acid diethylamide (LSD) by Sandoz chemist Albert Hofmann triggered a boom in Western psychedelic research. LSD and psilocybin were explored as models for psychosis and later as treatments for alcohol use disorder and depression (Krebs and Johansen 2012; Rucker et al. 2016). Early studies initially found large treatment effects that generated excitement, but these were not replicated in later, more tightly controlled studies (Hall and Farrell 2021). Concerns were raised about methodological rigour, the inappropriate use and distribution of study drugs, and blurred boundaries between advocacy and evangelism by researchers like Timothy Leary (Kious, Schwartz, and Lewis 2022). By the mid-1960s, psychedelics had “escaped the lab” and become a countercultural phenomenon associated with youth rebellion and resistance against the Vietnam war (Dyck 2008). Sandoz ceased production of LSD and psilocybin in 1965, citing a lack of financial incentive to renew the expired patent and government pressure to curb widespread recreational use (Hoffman 1980). Psychedelics were prohibited in the United States in 1970 by the Controlled Substances Act and research funding evaporated, ending the last U.S. psychedelic trial in 1976 (Carhart-Harris and Goodwin 2017; Oram 2016). From the perspective of funders and institutions, psychedelic research turned from a legitimate pursuit of medical and scientific discovery to an expensive and politically subversive undertaking that carried significant risks to personal and institutional reputations.

Global academic, medical, and commercial interest in psychedelics has been reignited by promising clinical research (e.g., Ross et al. 2016) and driven by fierce advocacy on behalf of powerful interests. Early research relied on philanthropic support, but the field has since received funding from government, multinational pharmaceutical companies, and smaller biotechnology startups (Phelps, Shah, and Lieberman 2022). Basic research has used psychedelics to explore consciousness, information processing, and brain network connectivity (Yaden et al. 2021a, b). Clinical trials have studied psychedelics as treatments for diverse indications, including depression, post-traumatic stress disorder (PTSD), anxiety, eating disorders, addiction, and distress associated with end-of-life diagnoses (Borgland and Neyens 2022; Wheeler and Dyer 2020). Whilst the term “psychedelic medicine” can be applied to the use of psychedelics in any health or traditional medicine context, the form predominately being trialled in western medicine is *psychedelic-assisted psychotherapy* (PAT). In PAT, one or more high doses of a psychedelic drug are administered within a programme of therapeutic sessions that prepare participants and help them to derive meaning from the psychedelic experience. Psilocybin therapy for treatment resistant depression and MDMA(3,4-Methylenedioxy methamphetamine) therapy for PTSD are the most advanced drug-indication combinations in terms of evidence and regulatory change. However, only MDMA is supported by published phase three clinical trial evidence; one trial with ninety participants (Mitchell et al. 2021), and one with 104 participants (Mitchell et al. 2023). Psychedelic research is nascent, and the history of modern drug development shows that promising results in small samples of selective populations are often not replicated in larger studies (Ona et al. 2022). Researchers also face challenges in accounting for confounds such as inadequate blinding, non-standardized psychotherapeutic techniques, and variable therapist–patient rapport (Muthukumaraswamy, Forsyth, and Lumley 2021; Hovmand et al. 2023).

Despite the need for caution in extrapolating early results, public enthusiasm is growing, described by some as a “hype bubble” in need of deflation (Petranker, Anderson, and Farb 2020; Yaden, Potash, and Griffiths 2022). Commentators, including researchers involved in the first wave of psychedelic research (i.e., Yaden, Yaden, and Griffiths 2021b), have raised concerns that current research risks repeating the exuberance and uncritical optimism of the 50s and 60s that contributed to the shutdown of the field. Policymakers are beginning to respond to public pressure to reform drug policy. Since 2020, Oregon and Colorado have legalized the provision of non-medical psilocybin services (Smith and Appelbaum 2022; Jeffries 2022). From July 1, 2023, Australia became the first country to federally legalize the prescription of psilocybin and MDMA in limited circumstances (TGA 2023) despite little psychedelic research having been completed in Australia (Gardner et al. 2019). The first clinical trial of psychedelic-assisted therapy in Australia began in 2019, and government funding of psychedelic research did not occur until 2021, with the award of AUS$15 million from the Medical Research Future Fund (MRFF). The decision by the Therapeutic Goods Administration (TGA) to partially reschedule psilocybin and MDMA was therefore surprising, even for those who had advocated for the change (Haridy 2023). The announcement was made only eight months after the TGA decided not to reschedule psilocybin and MDMA, based on public consultation and an independent expert panel report (TGA 2021). This was largely the result of strategic lobbying of the TGA by a well-connected philanthropic organization (Blau and Thompson 2023). As such, Australia serves as a salient case study of the receptiveness of regulatory agencies and policymakers to messages from advocacy groups that psychedelics are safe, viable, and profitable therapeutic agents that address unmet needs in the community; messages that both proponents and critics argue are premature and misleading (Hall and Humphreys 2022; Rossell et al. 2023). Premature adoption of technology can result in high profile harms that ultimately undermine innovation. As such, the Australian experiment represents a crucial juncture to understand what makes for ethical psychedelic innovation that maximizes its potential benefits whilst preventing undue harm in the community.

Bioethics scholarship on psychedelics is growing but has not kept pace with the rate of innovation and regulatory change. Issues addressed include gaining informed consent to an ineffable and potentially transformative psychoactive experience (Smith and Appelbaum 2022; Smith and Sisti 2020), the implications of models of the therapeutic mechanism that centre on increased suggestibility (Villiger and Trachsel 2023), harms arising from patient–therapist interactions during dosing (McNamee, Devenot and Buisson 2023), the need for culturally and racially inclusive research and practice (Michaels et al. 2018; Smith et al. 2022), and the appropriation of Indigenous knowledge without acknowledgement, consultation, or compensation (Celidwen et al. 2023; George et al. 2020). Psychedelic medicine presents novel ethical challenges as well as new ground for familiar debates. These are often dealt with in the literature as discrete issues. Exceptions include Spriggs and colleagues’ (2023) ARC framework, which presents Access, Reciprocity, and Conduct (ARC) as three pillars for evaluating and supporting ethical decision-making and practice in psychedelic medicine. The ARC framework is intended to be a site of collaboration and community feedback, highlighting the opportunity and need to engage stakeholders to support normative conclusions. However, beyond this emerging psychedelic-specific framework, there is also an opportunity to connect psychedelic medicine to existing ethics initiatives in science and technology, thereby supporting shared understanding between stakeholder groups, sectors, and disciplines and capitalizing on shared advances.

Responsible Research and Innovation (RRI) is a European Union research policy framework for ethical and sustainable technology development, that advocates for a collaborative relationship between researchers and societal actors from the outset of development (von Schomberg 2013). RRI recognizes a “responsibility gap” wherein governance cannot keep pace with innovation, because innovation inherently creates novel issues for ethicists, policymakers, and regulators to deliberate on, and these issues may not become apparent for some time after the innovation is implemented. The proposed alternative is seeing governance as part of the innovation process, and the collective responsibility of all actors involved in that process (Owen et al. 2013). Scholars have outlined the factors, goals, and processes that support the development of ethically acceptable, sustainable, and desirable technologies and/or practices. Stilgoe, Owen, and Macnaughten (2013) outline four dimensions of responsible innovation: *anticipation*, i.e. asking “what if?” in light of what is known, likely, and possible; *reflexivity*, i.e. the ability to reflect on one’s own activities, commitments, knowledge, and subjectivity; *inclusion*, i.e. the incorporation of a variety of actors into the innovation process whilst being conscientious of the power differentials at play; and *responsiveness*, i.e. the capacity for research and innovation to adapt in response to circumstances and the values of stakeholders. RRI recognizes that researchers are critical actors in the innovation ecosystem, whose experiences, values, and aspirations shape how intervention efforts aimed at addressing ethical and social issues are received and implemented. Psychedelic researchers are an expert and powerful stakeholder group, responsible for creating the evidence base for policy change and practice guidelines. They’re also key contributors to the public narrative, by promoting their own research and commenting on others’, responding to regulatory changes (e.g., Rossell et al. 2023), and lending their credibility and expertise to industry, advocacy groups, and media.

Previous interview studies of innovators have identified ethically salient information and views not otherwise represented in the literature. This includes topics such as how scientists view and navigate the presence of vested interests in alcohol research (McCambridge and Mitchell 2022), how they conceptualize their role and social responsibilities (Wäscher, Biller-Andorno and Deplazes-Zemp 2020), the precariousness of working outside the approval of traditional institutions (Guerrini et al. 2022), ethical and practical challenges to dominant positive narratives of citizen science (Riesch and Potter 2013), and concerns about appropriate use of evidence by policymakers and the boundaries between the roles of scientists and public figures during the COVID-19 pandemic (Colman et al. 2021). This body of empirical work, whilst small, has produced valuable information regarding scientists’ perspectives on their work, roles, and responsibilities, and social context that influence them. As such, it provides a data-driven basis for ethical deliberation and focuses efforts for systematic improvement on collaboration between affected stakeholder groups without impinging on researchers’ sense of scientific autonomy (Carrier and Gartzlaff 2020). This framing of researchers as stakeholders has not yet been applied to psychedelic research, leaving a gap in the literature regarding the factors and concerns that have shaped and will continue to influence the innovation trajectory and ethics of psychedelic medicine. To address this gap, we aimed to explore Australian psychedelic researchers’ experiences and perspectives, with a view towards supporting responsible innovation in the field through the principles of anticipation, reflexivity, inclusion, and responsiveness.

## Methods

The methods for this study are reported in accordance with the COREQ guidelines for qualitative research (Tong, Sainsbury, and Craig 2007). Ethics approval for this study was granted by the Monash University Human Research Ethics Committee (Project ID: 25739).

### Study Design

This study takes an empirical bioethics approach (Davies, Ives, and Dunn 2015), informed by a critical realist paradigm, to support inter-disciplinary engagement with the findings. The use of this approach reflects the multidisciplinary strengths of the research team, who come from psychology (MB), medical sociology (JG), and neuroethics AC). We are located within an Australian tertiary research centre with a psychedelic research programme and have professional relationships with researchers working in this field, including some that were participants in the present study. This professional and social situation provided access to potential participants, benefitted rapport in interviews, and led to the development of specialized knowledge in the area. As such, we consider ourselves to be informed interviewers (Laudel and Gläser 2007). However, we do not conduct scientific or clinical research on psychedelics and are ambivalent about the future of psychedelic medicine. The first author is a female researcher, undertaking this work as part of a doctoral research project.

A qualitative interview design was chosen to capture and describe the experiences, opinions, and values of Australian researchers studying the effects of psychedelics in humans or in animal models. Interviews were semi-structured and allowed for participant-led lines of inquiry, as the study aims were exploratory and we did not assume that all salient questions could be identified a priori. Interview question topics included: the goals of the organizations or groups that researchers were connected to; ideal outcomes and barriers to achieving these goals; drivers of psychedelic research and innovation; institutional factors and dynamics in conducting psychedelic research; ethical concerns about psychedelic research and practice; and sources of evidence and expertise.

### Participants

The participants in this study were researchers located within Australian tertiary research centres or hospitals and involved in basic, theoretical, or clinical psychedelic research with a focus on human behaviour, consciousness, cognition, and/or health. Target organizations, research groups, and individuals were identified based on the authors’ knowledge of the field, online interest groups, and snowballing. Purposive sampling was used to gain a range of perspectives across organizations, disciplines, professional backgrounds, and career stages.

Nineteen researchers were invited via email to participate, resulting in fourteen interviews. To protect anonymity, given the small number of psychedelic researchers in Australia at the time, participant details such as area of research and specific organizational or institutional affiliations have been withheld. The sample included individuals from eight Australian institutions, who were working on five of the six registered clinical trials in Australia at the time of recruitment, plus trials in the pre-registration design phase, and preclinical research. Fifty-seven per cent of the sample were male. Participants had backgrounds in neuroscience, psychology, pharmacology, psychiatry, and the humanities, and held roles as trial investigators, researcher-clinicians, teaching academics, PhD candidates, therapist trainers, and members of psychedelic research advocacy organizations.

### Data Collection and Analysis

Interviews ranged from twenty-five to sixty minutes and were conducted from February to November 2021. At this time, there were six registered psychedelic clinical trials in Australia, only one of which had treated participants. The day before the first interview a pool of AUD$15 million of Australian government funding was announced for psychedelic research. Since 2021, accounts of abuse and/or adverse outcomes associated with clinical trials of psychedelic therapy have become more widely acknowledged (e.g., McNamee, Devenot, and Buisson 2023), as have accounts of aggressive lobbying for legislative change (Blau and Thompson 2023). However, these accounts were not publicly recognized at the time of interviews. Interviews were conducted via Zoom by the first author, postgraduate provisional psychologist with training and experience in qualitative methods and both research and clinical interviewing, who identifies (and presents as) White and female. All participants gave informed consent to the interview and the use of their data in analysis and communication of results. Audio-recorded interviews were transcribed by either MB or a professional transcription agency. The interview schedule was piloted on the first participant and adjusted afterwards to improve the clarity and efficiency of the questions. Participants were invited to provide feedback on questions and informed of their right to view their transcripts for correction (this occurred in one case).

The dataset was thematically analysed using NVivo (QSR International) in an inductive manner with respect to the research questions (Braun and Clarke 2019). Reflexive thematic analysis was suited to capturing both the concrete (e.g., challenges faced in conducting psychedelic research) and speculative (e.g., ideal outcomes for psychedelic research) aspects of the study aims. The data was initially approached inductively wherein patterns of meaning and interest were coded, reflected on, and incorporated into the analysis. Field notes were made during interviews and referred to during the open coding phase. After open coding, the research questions and the literature were used as criteria to deduce the avenues for analysis that were most likely to be impactful, novel, and important to the aims of the study (Byrne 2022). The authors’ immersion in psychedelic research via working relationships and participation in community groups allowed for triangulation of the interview data with observed discussions between participants and other voices in the field. Data collection was concurrent with analysis and concluded when additional interviews produced minimal new lines of inquiry relevant to the research questions or divergent information in relation to established codes, and sufficient information had been obtained to produce a rich account of the themes (Saunders et al. 2018). Two versions of the thematic structure were produced by MB and then refined by all authors with reference to the dataset throughout the writing process, before the final themes were agreed upon. The present set of themes is one of two produced from the dataset, with the other examining researchers’ understanding of the future of psychedelics through the concept of promissory technology identities (Barber, Gardner, and Carter 2023).

## Results

Three themes were constructed through the analysis. These were: 1) coming out of the shadow of the 1960s; 2) challenges and affordances in engaging stakeholders, with subthemes relating to conservative and polarized clinicians, international mentorship, and psychedelic community; and 3) growing pains in innovation and translation, with subthemes relating to pace of change and hype, equity and accessibility, and accreditation and translation.

### Coming Out of the Shadow of the 1960s

When reflecting on psychedelic research, participants frequently referred to the mid-century psychedelic boom, the “War on Drugs,” and the recreational and countercultural uses of psychedelics. Participants described a conflicted relationship between contemporary and mid-century research. The expertise, knowledge, and promise generated by earlier research had laid the foundations for contemporary work but also cast a shadow of over-enthusiasm, poor-quality research practices and lessons of “what not to do” (Clinical researcher 2). Perceived biases against psychedelics, held by gatekeepers, the public and researchers themselves, had shaped many participants’ experiences of working in the field:

… people with a more conservative attitude still think that psychedelics are incredibly dangerous or toxic, or, if you use a psychedelic, you’re going to go and jump out a window or kill someone … I’ve come across psychiatrists who continue to have a lot of stigma towards these things, that are concerned about their professional reputations if we were to bring these kinds of trials into hospitals. So, I think that’s definitely a barrier. (Clinical psychologist 1)

Several participants told stories, either personal or those of colleagues, of being blocked from applying to conduct psychedelic research. In the quote below, as in the one above, these blocks were attributed to attitudes toward psychedelics, therefore contributing to the sense in the data that contemporary psychedelic research had needed to contend with or push past its own history to be considered a safe and viable “science.”

It was like a denial of scientific research that really wasn’t hurting anybody, it was very safe and yet we weren’t allowed to do it, because of that stigmatization and the association with illegal drugs, which was very deeply held negative beliefs. That was the frustrating thing. The science was being stopped by that. (Psychiatrist 1)

However, the view that negative bias had impacted psychedelic research in Australia was not ubiquitous. Two participants expressed the view that obtaining funding and navigating the regulatory environment associated with highly scheduled drugs had been barriers to research but not made unduly difficult compared to other projects:

I didn’t think it was blocked as such. I think it was just hard to get funding. It’s hard to get funding for all sorts of research, so I didn’t feel like any degree of advocation was going to help too much. (Neuroscientist 2)

Whether the slow start to psychedelic research in Australia was attributed to historically rooted attitudes or not, the “coming out of the shadow” aspect of this theme was informed by the observation made in most interviews that the cultural identity of psychedelics and psychedelic research was changing over time, in both the public and academic spheres, galvanized by popular media. Michael Pollan’s book *How to Change Your Mind* (2018) was mentioned in most interviews:

There were key pivot points … the New Yorker article and then the book by Michael Pollan who was a writer of respect a long time prior to his interest in psychedelics … quite a lot of people sort of lost their fear of stigma and backlash and then started to speak up. (Pharmacologist 1)

Though positive media was acknowledged as a facilitator of psychedelic research overcoming attitudinal and/or funding barriers, a subsequent concern expressed by participants was the vulnerability of the field to being set back or shut down again due to malfeasance or sensationalism—a potential repeat of the past in some cases. To protect ongoing psychedelic research against this perceived heightened scrutiny, participants pointed to different solutions. Clinical trial researchers described prioritizing rigour and adherence to scientific principles, as well as the safety, training, and careful selection of people involved in trials. Participants involved in preclinical and basic science studies highlighted the need for preclinical animal or healthy participant research to pre-empt potential controversies in intervention trials, as illustrated below:

The concern is that something will happen in one of these clinical trials, and the participant will have some contraindication that we don’t really know about yet … I guess everybody’s underlying concern is that that will set the field back, again … the risk of an adverse event is just around the corner. And because of the sensationalized media hype, it’s not going to go unnoticed if something happens*.* (Preclinical researcher 1).

Though views were variable between participants, the shadow of 1960s psychedelic research appeared to have enduring impacts on their experiences as psychedelic researchers, and their view of the potential for media and public discourse to impact psychedelic research to either the benefit or detriment of the field ongoing.

### Challenges and Affordances in Engaging Stakeholders

In reflecting on the story of Australian psychedelic research and looking to its future, participants described the potential benefits and challenges in engaging stakeholders and their goals to stabilize the field and protect it against risky, underqualified, or over-enthusiastic actors. Relationship building and values of patience, tenacity, and humility, were key features in many participants’ narratives of the growth of research:

The story has been one of a huge amount of pro-bono effort over the last couple of years in building relationships with people overseas … and in building bridges here internally in Australia … on the shoulders of the giants overseas who have done so much to make this happen … Initially they were small, unfunded trials, all pro-bono, with various compromises … to the point now where it feels really well-aligned and well-funded*.* (Clinical researcher 1)

In the context of discussing barriers and facilitators to psychedelic research, three stakeholder groups were discussed most often: clinicians, the international psychedelic research field, and the non-academic psychedelic community.

#### Conservative and Polarized Clinicians

Clinicians were generally characterized as conservative and resistant to change, and therefore a potential barrier to the progress of psychedelic medicine. However, the approval of this group was also presented as a potential affordance, resulting in the need for strategic engagement with clinicians to advance the goals of psychedelic research:

… our strategy is to involve the medical community in this research to the extent that they recognize the merits and are prepared to consider these alternatives … the medical community is really the first line of defence that we need to work with. (Pharmacologist 1)

To this end, participants emphasized the need to strike a balance between appearing cautious and risk-averse whilst also advocating for the potential benefits of psychedelics. Participants often referred to the power of “the science” in engaging ambivalent or negatively biased groups:

Get really schooled up in the science of it before you start opening your mouth in a public way. Being sensible. Not exaggerating the benefits. Not downplaying the risks. Not exaggerating the risks and downplaying the benefits. It’s that kind of tension between the two. Just carry the science wherever you go. (Clinical psychologist 1)

Some participants framed this strategic engagement as a linear process of accruing trial evidence and then actively promoting it to the broader clinical field:

Conservative psychiatry and psychology have to listen because the early results are good, and they’re being pushed into listening. They can’t avoid not listening, because those who are doing the research and talking about it are making a lot of noise. (Psychiatrist 1)

However, other participants, both neuroscientists and clinicians, questioned the focus and quality of the science currently being used to advocate for psychedelics. One participant felt that uncritical advocacy had contributed to the polarization of clinical opinion regarding psychedelics:

We’ve got a group of advocates who believe that this is going to be the panacea for all human ill and then we’ve got a considerably larger and potentially much more powerful group of people who say this is dangerous, this is not particularly effective, this is untested, this is fundamentally moonshine*.* (Psychiatrist 2)

This polarization was described as a “see-saw between scepticism and hope*”* (Preclinical researcher 1) driven by distrust of over-optimistic claims paired with strong desire for a novel, effective intervention for intractable psychiatric disorders. From the perspective of interviewees, this suggests that strategies for engaging clinical stakeholder groups that are insensitive to scepticism may inadvertently perpetuate some of the barriers to gaining the affordances of their involvement and approval.

#### International Mentorship

Participants discussed the affordances of connection to the international psychedelic research community; namely guidance and expertise, which was seen as lacking domestically. When asked to characterize the Australian field, participants described Australia as “behind” (Clinical psychologist 1), “not where they’re at” (Clinical psychologist 3), and “dependent on overseas results” (Psychiatrist 1; Note: interviews occurred prior to the TGA rescheduling of MDMA and psilocybin). However, participants also described a growing independent voice for Australian psychedelic research signalled by a significant government investment in the field (i.e., the MRFF grants):

I think [the MRFF funding] gives a legitimacy to the field in some ways … the people that I’ve heard that are applying for MRFF … are all highly skilled clinical researchers with a goal to do good research … it certainly makes what I’m doing seem less crazy … I think it’s all positive in terms of putting Australia on a level playing field with the world in that respect. (Preclinical researcher 1)

In the context of growth in the Australian field, losing touch with international mentorship was seen by some participants to pose a risk to the stability and longevity of research:

I see this time in Australia as being exciting and precarious for the next couple of years, like a bit “wild-westy” … all kinds of people trying to jump into the space and dominate, or take control, and very little expertise, very little connection or bridges being built to the people overseas who know what they’re doing. (Clinical researcher 1)

In a period that participants characterized in terms of increased funding, interest, and opportunity in Australian psychedelic research, this participant expressed concern that new research actors would not feel the need to engage with international mentorship, and would therefore risk losing out on the perceived guidance and expertise afforded by this stakeholder group.

#### Psychedelic Community

In discussions regarding sources of expertise and evidence in psychedelic research, several participants identified the psychedelic “community” as an influential stakeholder with positive affordances. In this context, “community” refers to groups with an interest in studying or practicing with psychedelics, often with a healing, ethnobotanical, spiritual, or personal growth focus:

There’s definitely a grass roots, almost, psychonautic community. Those who are taking these compounds, yes to grow and to explore and who need the guidance and community around that, and holding and knowledge sharing, which I think is really important. They all obviously believe and very much support the research community which is more focused on academics. (Clinical researcher 3)

This group was framed as generally supportive of and integrated with research, and knowledge derived from these groups was seen by some participants as influential and of benefit to academic research:

For a long time before there was a thriving research network of psychological scientists interested in psychedelics in Australia, there’s been non-academic conferences and meetings of psychedelic enthusiasts and there’s quite a lot of underground knowledge within those networks. I think that feeds into the scientific and academic work and it’s quite an important source of information actually*.* (Cognitive neuroscientist 1)

In comparing the three key stakeholder groups identified in discussions with participants, the affordances of engaging international mentors and the psychedelic community appeared to be centred on their perceived expertise, whereas the affordances of engaging clinicians appeared to be centred on their potential to otherwise be a barrier to progressing psychedelic research within the medical system. Furthermore, participants also noted “tension in the Australian community around the medicalisation of this” (Clinical psychologist 2) and that some factions of the psychedelic community may not support research if it is at odds with or limits the broader uses of psychedelics. This is suggestive of potential conflicts between the goals of stakeholder groups and the strategies needed to engage these groups.

### Growing Pains in Innovation and Translation

“Growing pains” captures ethical tensions and arising from the rapid expansion of the psychedelic field in Australia over several years, including pace of change and hype, equity and accessibility, and workforce development and accreditation.

#### Pace of Change and Hype

In describing Australian psychedelic research, participants noted excitement for change, pressure to move quickly on projects, and concern about the speed and overhyping of developments in knowledge and policy. For some, this scenario felt like a welcome reward for patient advocacy and a sign of imminent translation:

We went through a phase of advocacy, which was a bit difficult and interesting, got lots of knock-backs. Then we finally got the research going … It’s blossoming. The Australian Government’s given $15 million … The next stage will be the implementation of the clinical practice. Even though, as a researcher, I’m supposed to say, well, we’ve got to wait for the results, I’m an impatient sort of person and I think the results are going to be good. I think that in the next two or three years there’ll be the implementation of clinical treatment using psychedelics. (Psychiatrist 1)

However, almost all participants, including those who spoke with the greatest optimism about psychedelic therapies and those who had described negative attitudes as a barrier to conducting psychedelic research, expressed concern about a pendulum-swing towards the overhyping of psychedelic therapies and “excessive evangelism” (Pharmacologist 1):

I think the field has been a little bit slowed down because of the panacea thinking … “we’ve got it, this is in the bag, we just need to like run it through the phase three trial and at the end of the line it’ll be done.” … These kind of very impressive long-term effects of a short psychedelic treatment programme are just unlikely to scale up. (Clinical researcher 1)

With the perceived growing ease of gaining funding and approval for trials, some participants expressed concern about the competency and intentions of newer researchers in the space compared to established, internationally mentored groups, and the impact this might have on the reputation and longevity of psychedelic research:

… there is a risk that people who might be less skilled or qualified or able to undertake these types of studies might start to do so because of the excitement and the hype and the money … my fear is that a couple of poorly designed studies, or a couple of a studies which result in bad outcomes for people might derail the initiative. (Psychiatrist 2)

Additional concerns regarding hype cited by participants included premature policy change and consumers seeking unapproved psychedelic therapies:

The more hype there is in the media about this, desperate people will do desperate things … I really worry about people then accessing these treatments in the underground environment in Australia because there are some good people working in the underground at the moment, but there are also some dodgy operators and it’s really Russian roulette. (Clinical psychologist 2)

#### Equity and Accessibility

In considering the growth and future of the field, a second theme of concern raised by participants was maintaining the integrity and safety of psychedelic therapy whilst ensuring equitable access for those who would benefit most:

… the question will be whether it’s going to be affordable … or whether it remains the preserve of rich people ... If it goes on PBS it’s cheap, but you can’t use the drug unless you support it with the psychotherapy, and the psychotherapy will be potentially quite expensive. (Psychiatrist 2)

From one participants’ perspective, the primary barrier to equitable access was that clinical research with psychedelics had primarily been designed for a privatized model of delivery:

I really foresee that it will become a niche thing, in these uber-deluxe spa type of treatments … it won’t get picked up in the public sector because it’ll be too expensive. It’ll be too time-consuming … to get it to a point where it’s sort of Medicare supported, we’ve got to move so far from where it’s currently being tested. There doesn’t seem to be willingness of the academic researchers to test a sort of shaved-down version of the current treatment. (Neuroscientist 2)

Another participant described the dilemma of researching a therapy in populations who may not have the means to access to it in practice:

[I] realized that the patients in my [clinical pharmacotherapy] trial were never going to be able to afford it, even if we showed it was effective … the same thing is going to happen with MDMA and psilocybin … obviously we can’t give everybody the access they need through clinical trials. But we need the clinical trials to get to a point where we can say whether or not these are useful treatments. (Clinical psychologist 2)

While most participants saw increasing industry involvement as inevitable, some also saw for-profits as a further threat to equity:

… there are always venture capitalists who kind of want to make a buck and profit out of an idea … we have to be really careful about how we roll this out … I’m just hopeful that we can make this an equitable and accessible treatment wherever it lands. (Clinical psychologist 2)

One participant suggested a collaborative approach to managing the ethical impacts of scaling up psychedelic medicine, drawing on the idea of the “community” coming together to determine standards for for-profit conduct:

Unfortunately, we are going to see for-profit companies moving in in the next five years most likely and I think that there are opportunities for that to be done … in ethical ways that encourage social benefits versus profit … I feel that community cohesion and/or a shared ethical pledge could have the role of—I wouldn’t necessarily say policing but bringing awareness to bad actors. (Clinical researcher 3)

Lastly, one participant expressed their confidence in clinicians to balance industry interests whilst progressing promising therapies:

The one good thing about medicine is that irrespective of what the company or the promoter says about a medicine or a drug … if people use something and it doesn’t work, they’ll drop it like a hot potato. Conversely, if they know that something does work … even if the PBS doesn’t allow it, people will still use it if it works. (Psychiatrist 2)

#### Accreditation and Translation

Complicated by the rapid pace of change, and integral to facilitating equitable access, was the issue of determining the necessary competencies for psychedelic therapists. Participants acknowledged the challenge in finding individuals with both psychopharmacological knowledge and psychotherapeutic skills:

… currently it’s mainly falling on psychiatrists. They might be the best person, but they also could not be, there could be some other ways of making it more accessible. (Clinical researcher 2)

One participant (Clinical psychologist 1) suggested that at least “five years” experience prior to training in psychedelic therapy was necessary. However, the requirements of any additional training, and the relationship to baseline professional qualifications, was unclear to some participants:

Some people think you need really intensive training to provide psychedelic assisted psychotherapy. Other people think, as for example, clinical psychologists, you have the training to provide therapy, you don’t need that much … I would say at least some training needs to be involved because it is quite different to therapies like traditional cognitive behaviour therapy, for example. (Clinical psychologist 3)

Finally, participants generally expressed a desire to see psychedelic therapy regulated using existing systems, such as health regulators (e.g., the Australian Health Practitioner Regulation Agency) and the codes of ethics of various professional bodies in mental health.

## Discussion

The results capture the tensions felt by Australian psychedelic researchers in a field moving from a small group of committed individuals with shared values and worldviews, to a larger entity involving commercial interests, scientific and healthcare institutions, government regulation, public engagement, and reduced individual control over the narrative and direction of progress. The transition was characterized by researchers seeking differentiation from psychedelics’ past, support from traditional and non-traditional stakeholders, a place at the table of international research, and negotiation of the legitimate voices, evidence, and providers of psychedelic therapy. Participants articulated a tension between the slow, evidence-based approach needed to responsibly innovate psychedelic medicine and engage conservative stakeholders and the benefits of the growing momentum in unlocking opportunities and funding to advance research. Though psychedelic research faces some unique challenges, this is a process that all innovations must go through (Greenhalgh and Papoutsi 2019). RRI has played an increasingly prominent role in guiding neuroscientific and medical innovation (Stahl et al. 2021). By applying it as a lens to psychedelic medicine we intend to link the developing field of psychedelic ethics into a framework of theory, policy, and practice based on multidisciplinary collaboration that has scope to inform governance both in the clinical applications of psychedelics and the potential non-medical developments (e.g., wellness, neuro-enhancement; Wexler and Sisti, 2022). By applying the dimensions of reflexivity, anticipation, inclusion, and responsiveness to our findings, we connect the challenges raised by participants to targets for RRI-informed initiatives that support the potential positive impact of psychedelic medicine on community well-being.

### Anticipation

Anticipation is asking the question of “what if?” in light of what is known, likely, and possible. Our findings are illustrative of the tensions and limitations of anticipating the potential impacts of innovative mental health technologies. Participants drew on the history of psychedelics as evidence of the potential for public and institutional backlash and the development of this current wave of research in a direction at odds with their personal ethics and societal values. However, they also acknowledged limits in the knowledge used to make these predictions (e.g., a lack of preclinical evidence of contraindications) and expressed concern about the lack of control over how other stakeholders might use research evidence to suit their own agendas. Some expressed a lack of faith in industry and the health system to protect the interests of consumers and maintain the integrity of psychedelic therapies. Issues raised included balanced media reporting, the impact of hype on clinician attitudes and public expectations, equitable access to PAT, and responsible industry involvement.

The core of these issues is in anticipating the impacts of producing, reporting, and using scientific evidence, on policy change, accessibility, treatment integrity, and inclusion of minority and vulnerable populations. Participants were aware of the impact of science communication and described “the science” as a tool for engaging stakeholders by evoking conservative values of caution, rationality, and dispassion whilst bolstering optimistic statements about psychedelics. However, some participants also acknowledged that “the science” itself is diverse in focus, quality, and translatability, and not always disseminated or received with an appropriately critical lens. Researchers are not always experts in healthcare policy, politicians are not always science experts, and consumers should be consulted regarding their needs and preferences, minding the difference between testimony-based advocacy and clinical evidence. In a focus-group study, Loroño-Leturiondo and Davies (2018) found that scientists vastly attributed responsibility for the outcomes and impacts of science communication to themselves, but as the authors argue, this is likely more idealistic than a literal account of practice. Embracing co-design and a collaborative, inclusive, multi-stakeholder approach to evidence production and dissemination diffuses this responsibility, promotes anticipation of the needs of society, establishes priority-driven research agendas, empowers consumers, and designs research that dovetails with healthcare infrastructure (Slattery, Saeri, and Bragge 2020).

### Reflexivity

Reflexivity is the ability to reflect on one’s own activities, commitments, knowledge, and subjectivity. The results illustrate factors that promote and impede reflexivity on behalf of the research community, and provide insight into how input from other stakeholders might be received. The history of psychedelic research appears to loom large in the minds of Australian researchers and shapes their perspectives on what they envisage as responsible psychedelic medicine. Participants’ concerns about overhype were expressed alongside unease regarding the vulnerability of the field to backlash and premature discontinuation, evidenced by pointing toward psychedelics’ tumultuous history. According to our interviewees, this had prompted greater conscientiousness in designing and conducting research, which lends itself to responsible innovation. However, as highlighted by Appelbaum (2022) and by participants in this study, stringent exclusion criteria and intense therapeutic support decrease the chance of an adverse event and maximize positive outcomes but also result in impractical protocols that are expensive to implement and may not be generalizable to the broader population. The rapid pace of translation from evidence to policy in psychedelic medicine at present means that systematic trial-and-error with at-risk or excluded populations is likely to occur in the community through expanded access, Authorised Prescriber schemes, and/or legalization for non-medical use, and increased availability in community may hinder the collection of valuable safety and efficacy data through clinical trials, which have more stringent reporting standards and scope for assessing and controlling bias than real-world data or prescriber reporting (McGuire, et al. 2023). Excluding riskier or more challenging populations from in-human clinical trials may protect psychedelic research from controversy but poses additional risks to consumers if policy progresses regardless and may ultimately undermine innovation. The collaborative and systematic deflation of over-inflated expectations by stakeholders, including researchers, would produce a more sustainable and effective therapy (Yaden, Potash, and Griffiths 2022). In this case, bad press, if it represents honesty, transparency, and a willingness of researchers to take calculated risks in trials rather than letting those decisions occur blindly in community practice, can be good press.

Another area for considering the role of reflexivity, is in the perceived conservatism of the medical community. Survey and interview studies of psychiatrists, psychologists, and counsellors have found generally favourable attitudes towards evidence-based, medical uses of psychedelics, but also concern regarding drug diversion and the potential for psychiatric harm if used without medical supervision (Barnett, Siu, and Pope 2018; Davis et al. 2022; Hearn, Brubaker, and Richardson 2022; Page et al. 2021). This highlights a core tension for psychedelic research noted by participants in this study: delineating the medical agent from its cultural, recreational, and alternative medicinal uses (Andrews and Wright 2022). As Noorani (2020) argues, using clinical research to institutionalize definitions of “proper use” versus “abuse” of psychedelics sidelines knowledge and practices drawn from the social and cultural history of psychedelic use and reinforces politically motivated inconsistencies in global drug policy (Nutt et al. 2007). On the other hand, clinical “conservatism” regarding the potential dangers of widespread nonmedical use of psychedelics is not entirely unfounded. The legalization of cannabis in many United States states following changes in medical use policy has been associated with increased incidence of cannabis use disorder and a concurrent increase in the potency of cannabis with a decrease in public perception of its harms (Chiu et al. 2021; Smith and Appelbaum 2022). Gaining widespread acceptance for psychedelics with clinicians may be the next hurdle, but the safety and social justice consequences of this kind of acceptance should be considered. This requires reflexivity and transparency regarding stakeholders’ goals for policy change and the normative assumptions they hold about psychedelics’ use in various contexts.

### Inclusion

Inclusion is the incorporation of a variety of actors into the innovation process whilst being conscientious of the power differentials at play. The growth of psychedelic medicine will require the involvement of a growing number and diversity of actors. In discussing ethical concerns, some participants referred to bad faith or inexperienced actors whose actions might lead to inefficient research, harms to participants, and the threat of discontinuation of psychedelic innovation. There have been examples of poor research practices, both historic (Strauss, de la Salle, and Sloshower 2021) and contemporary (McNamee, Devenot, and Buisson 2023) that have done harm to consumers and damaged the reputation of psychedelic research. Concern has also been raised about the commercialization of psychedelic medicine driving premature policy change and resulting in an inequitable distribution of benefits (Smith and Appelbaum 2022). Identifying harmful practices and misuse is an important part of protecting consumers. In turn, being able to classify certain actors as “good” or “bad” affords psychedelic research some protection if an adverse event is sensationalized. However, if done uncritically, harms may be over-attributed to specific people and protocols rather than the drugs themselves. As Australia moves into an era of increased diversity in psychedelic research and policy change that makes way for community practice, the potential for user error will need to be incorporated as part of the risk profile of psychedelics, rather than as an explanatory caveat to adverse events. Williams and colleagues (2021) discuss the “fractionation” of the Australian psychedelic research community as a potential barrier to growth and translation. Whilst the authors focus on fractionation driven by disagreements about the pace of change and professional divisions, an uncritically applied narrative of “good” and “bad” actors may also result in a fractionated field that precludes deliberation regarding the use, safety, and efficacy of psychedelics in a range of settings and experience levels. Fostering an inclusive research environment may look like an inclusive, stakeholder engagement approach to trial design, policy, and governance that aims to capture the ideals, norms and goals of a wide range of actors (current and future) who develop, use or invest in psychedelic medicine could help to address the “fractionation” of the field through shared problem solving.

### Responsiveness

Finally, responsiveness is the capacity for research and innovation to adapt in response to circumstances and the values of stakeholders. Researchers in this study were concerned about equity and accessibility and the potential that psychedelic research would only benefit those with the capacity to pay for psychedelic therapy, currently estimated at AUD$25,000 per course of treatment (Chrysanthos 2023). A perspective from our findings was that research, not industry, was responsible for facilitating accessible psychedelic medicine by designing translatable protocols that respond to the specifics of the mental healthcare system. This highlights the need for research that not only aims to find results but is responsive to the values and conditions of the society it hopes to translate those results into. A large proportion of this estimated cost is attributable to clinician time and expertise (Mihalopoulos, Langmead, and Chatterton 2023). Professional mental health bodies and industry will need to come together with researchers to examine the necessary components of safe and effective psychedelic-assisted therapy, and whether this requires changes to current clinical practices. Clear guidelines are needed to determine which health professionals are qualified to provide the non-directive and theoretically flexible framework for psychotherapy currently used in trials (Cavarra et al. 2022). It is also worth anticipating what professional practice with psychedelics might look like, including who is likely to seek training in psychedelic-assisted therapy, for what amount of compensation, and the settings in which they envision themselves working. Together, this could begin to address issues of unregulated accreditation pathways and manage consumer expectations about the availability and accessibility of psychedelic therapies, if approved.

### Limitations

Design and data collection for this study occurred in 2020–2021. This study captures the perspectives of highly influential academic researchers but not private pharmaceutical researchers, as this was not happening with psychedelics in Australia at the time. Industry researchers may hold different attitudes, particularly when it comes to concerns about the role of for-profit companies in ensuring equitable access to psychedelic therapies. Since 2021, Australian psychedelic research has grown substantially, from six locally registered clinical trials to over fifteen as of late 2023. As such the individuals interviewed here could be characterized as “early” Australian psychedelic researchers. We acknowledge that there are likely to be many perspectives not represented in our data, and that the perspectives of those sampled are likely to have evolved in response to developments in the field since interviews were conducted. Future research into the perspectives of industry representatives, non-research advocacy groups, venture capitalists, and community groups would also be beneficial.

## Conclusion

We explored psychedelic researchers’ experiences and concerns and viewed these concerns through an RRI lens, emphasizing anticipation, reflexivity, inclusion, and responsiveness. The focus on Australian research here serves as a case study of factors and issues that may emerge in other jurisdictions, especially those where psychedelic research has only recently emerged. Participants described goals to develop psychedelics in a way that prioritizes consumer safety and accessibility and reduces the likelihood of a premature halt to research, whilst allowing for creative innovation in mental health. Issues raised by researchers that could benefit from RRI-informed stakeholder collaboration include equitable access, responsible and sustainable industry involvement, productive research agendas, balanced media reporting, and clear accreditation pathways for psychedelic therapy training. Responsible reporting of evidence, embracing transparency, risk-taking within the relative safety of clinical trials, and critically reflecting on what makes for “bad“ or “good” actors in relation to psychedelic medicine, are areas of growth for the field and its stakeholders to focus on. Importantly, what this study highlights, is that the consequences of a new technology, both positive and negative, are not researchers’ responsibility alone. There is great scope for a collaborative, locally tailored approach to the development and translation of psychedelic medicine that protects consumers and advances mental health treatment for those who need it most.

## Data Availability

Due to the nature of the research, data for this study is not publicly available as it contains information that could be used to identify participants.
